# Corrosion and Corrosion Protection of Additively Manufactured Aluminium Alloys—A Critical Review

**DOI:** 10.3390/ma13214804

**Published:** 2020-10-28

**Authors:** Reynier I. Revilla, Donovan Verkens, Tim Rubben, Iris De Graeve

**Affiliations:** Research group of Electrochemical and Surface Engineering, Department of Materials and Chemistry (MACH), Vrije Universiteit Brussel, Pleinlaan 2, 1050 Brussels, Belgium; Donovan.Verkens@vub.be (D.V.); tim.rubben@vub.be (T.R.); Iris.De.Graeve@vub.be (I.D.G.)

**Keywords:** metal additive manufacturing, aluminium alloys, corrosion behaviour, microstructure, corrosion protection

## Abstract

Metal additive manufacturing (MAM), also known as metal 3D printing, is a rapidly growing industry based on the fabrication of complex metal parts with improved functionalities. During MAM, metal parts are produced in a layer by layer fashion using 3D computer-aided design models. The advantages of using this technology include the reduction of materials waste, high efficiency for small production runs, near net shape manufacturing, ease of change or revision of versions of a product, support of lattice structures, and rapid prototyping. Numerous metals and alloys can nowadays be processed by additive manufacturing techniques. Among them, Al-based alloys are of great interest in the automotive and aeronautic industry due to their relatively high strength and stiffness to weight ratio, good wear and corrosion resistance, and recycling potential. The special conditions associated with the MAM processes are known to produce in these materials a fine microstructure with unique directional growth features far from equilibrium. This distinctive microstructure, together with other special features and microstructural defects originating from the additive manufacturing process, is known to greatly influence the corrosion behaviour of these materials. Several works have already been conducted in this direction. However, several issues concerning the corrosion and corrosion protection of these materials are still not well understood. This work reviews the main studies to date investigating the corrosion aspects of additively manufactured aluminium alloys. It also provides a summary and outlook of relevant directions to be explored in future research.

## 1. Introduction

Metal additive manufacturing (MAM), commonly known as metal 3D-printing, is a process by which complex multifunctional metal parts are produced in a layer by layer fashion using 3D computer-aided design (CAD) models [1,2,3,4,5,6]. Several MAM techniques are available. They can be separated into two main groups: direct energy deposition (DED) methods and powder bed fusion (PBF) technology [7]. During direct energy deposition, focused thermal energy is used to fuse materials by melting as they are being deposited; while during powder bed fusion, thermal energy selectively fuses regions of a powder bed [7]. DED processes such as wire arc additive manufacturing (WAAM) and laser metal deposition (LMD) can generally be used on existing parts of arbitrary geometry with a relatively high deposition rate; however, the shape complexity is limited. This makes DED processes the preferred methodology for repairing or improving existing parts [8]. On the other hand, on PBF methods such as selective laser melting (SLM), selective laser sintering (SLS), and electron beam melting (EBM), the dimension of the pieces is limited and the starting substrate has to be a flat surface. However, they generally allow the fabrication of pieces with extremely high structural complexity at a relatively high level of precision. Among the several MAM processes, those utilizing a metal powder feedstock and a laser source to achieve the metal fusion are the most widely used [1,2,3,4,5,6]. From those, SLM is regarded as the most used and studied MAM method. This is not only because it allows a higher level of precision compared to other MAM techniques, but also because (in contrast to SLS) it allows the full melting of the material, and therefore the production of solid and dense metal parts in a single process (without the need to use binders and/or other post-process furnace operations).

Additive manufacturing is considered one of the enabling technologies for Industry 4.0 [9]. In particular, MAM presented a global market valued at € 2.02 billion in 2019 [10]. This included systems, materials, and services. MAM allows the near-net shape manufacturing of geometrically complex parts such as lattice structures and 3D structures with undercuts or cavities, which is why this technology has found numerous applications in industries such as medical implants, energy, aerospace, and automotive. As an example in aerospace applications, MAM has made possible the re-design of complex fuel injector nozzles (commonly requiring the assembly of more than 20 parts) in a single operation [11,12], as well as the re-design of several other complex engineered parts, resulting in considerable cost and weight reduction. In aerospace, as well as in the automotive industry, MAM is also actively used in prototyping and the fabrication of custom tooling.

Nowadays, a great number of metals and alloys can be processed by additive manufacturing techniques, depending mainly on the availability of the raw materials as metal powders or metal wires [5]. Amongst these, aluminium alloys are of great interest for applications requiring high strength and stiffness to weight ratio, good wear and corrosion resistance, and recycling potential, which is why they are attracting increasing attention of the automotive and aerospace industries [12,13]. The most common Al-based alloys processed by additive manufacturing (AM) either for commercial use or for research purposes are [5,14,15]: AlSi12, AlSi10Mg, AlSi7Mg0.6, AlSi9Cu3, AlSi5Cu3Mg, AA1050, AA2017, AA2024, AA2219, AA6061, AA7020, AA7050, AA7075, and AA5083; next to proprietary industrial alloys like Scalmalloy. From those, Al-Si alloys, and more specifically AlSi10Mg (followed by AlSi12), are undoubtedly the most investigated and commercially used additively manufactured Al-based alloys. These materials, particularly relevant for light-weight and high-strength applications, are widely used for aluminium casting due to the proximity to the eutectic composition (~12.5% Si) [16]. Therefore, they are relatively easy to process by laser applications, which are known to lead to a small solidification range [17]. Additionally, minor additions of magnesium (0.3–0.5 wt.% Mg) are known to induce hardening of the alloy by forming Mg_2_Si precipitates upon natural or artificial ageing treatments [18]. However, the actual formation of these precipitates on additively manufactured Al-Si parts is still a topic of discussion.

Due to the special conditions of the MAM processes (namely that the metal powder used is already pre-alloyed, and the melting occurs in small pools that rapidly solidify), a very fine microstructure with unique directional growth features far from equilibrium is achieved [1]. This distinctive microstructure, together with other special features and microstructural defects originating from the additive manufacturing process is known to greatly influence the corrosion behaviour of these materials. Sander et al. [19] and Kong et al. [20] reviewed the impact of these special features and defects on the corrosion performance of additively manufactured metals. These works also consider the main corrosion issues of several additively manufactured materials, including some studies on Al alloys. Zhang et al. [17] presented a review of Al-based alloys summarizing the microstructural characteristics and mechanical properties. Chen et al. [21] reviewed the main research studying the corrosion behaviour of selective laser melted Al alloys, classified/structured by the used technique.

The present work reviews the main studies to date investigating the corrosion aspects of additively manufactured aluminium alloys from a general perspective. Some of the defects and intrinsic issues from the additive manufacturing process that can influence the corrosion performance of these materials are discussed. Additionally, the most relevant studies and results concerning the effect of microstructure, heat treatments, Si content, surface roughness, and surface treatments on the corrosion behaviour of Al-based alloys are identified and discussed. Moreover, the main aspects concerning the behaviour of these materials to corrosion protection surface treatments such as anodizing are summarized and discussed for the first time. A summary and outlook of relevant directions to be explored in future works are also presented. The majority of the works investigating additively manufactured Al alloys was conducted on SLM materials, and a few studies were realized using SLS specimens. The microstructural characteristics and corrosion behaviours of the materials fabricated using those two methods are remarkably similar. Therefore, the use of “AM Al alloys” in this work refers, in general, to SLM and/or SLS Al alloys.

## 2. Influence of Defects in AM Specimens on Corrosion Resistance

Metal additive manufacturing presents, without a doubt, great potential to become a key manufacturing technology in several industries and daily life applications. One of the conditions still needed to achieve this goal is better control and understanding of the numerous macro- and micro-structural defects introduced by the special manufacturing conditions of these processes. These defects can greatly influence the materials’ performance against corrosion [22]. Further investigations are needed to better understand the effect of features such as porosity, surface roughness, and residual stresses, among others, on the corrosion resistance of these materials.

Metal additive manufacturing is, in general, a very complex process with a great number of operation parameters involved (for instance the laser/beam power, scanning speed, the spot size of the power source, hatch distance, layer thickness, powder flow speed in the case of DED processes, etc.). The effect of each process parameter in the final printed part cannot be seen independently of the rest. Instead, the energy density (E), which indicates the energy input into the material (see Equation (1)) and is equal to the laser/beam power (P) divided by the scanning speed (v), the layer thickness (d), and the hatch distance (h), was the quantity considered to evaluate the effect of process parameters on the properties of the final parts [23]. Previous studies have shown that the energy input is the determining parameter for defining and optimizing the “process window” during additive manufacturing [23]. A low energy input per unit length results in droplet formation and a bad wetting to the previous layer. On the other hand, a relatively high energy input per unit length causes distortions and irregularities due to big melt pool volumes and the balling effect. The specific process parameters and, therefore, the energy density used, are usually optimized per alloy type in each AM system to obtain high-density parts with the lowest possible level of porosity. Nevertheless, confirming the fact that MAM is a complex process, other researchers believe that the energy density cannot be the sole parameter analysed during the process optimization [24]. Other parameters such as the scanning strategy, laser spot size, and materials’ properties (i.e., thermal conductivity and reflectivity) should also be considered. Moreover, the researchers suggest that the laser power has a greater influence than the rest of the parameters, and therefore should also be considered independently [24]. Furthermore, Leung et al. [25] also demonstrated that the oxidation state of the metal powder could have a great influence on the formation of defects in the printed parts.

(1) $$E=\frac{P}{v\times h\times d}$$

**Figure 1:**
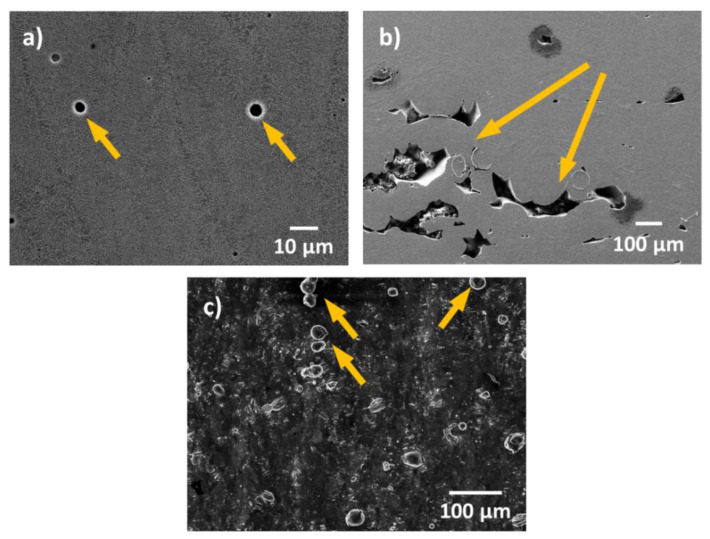
Secondary electron image representing defects that can be present in additively manufactured metal parts: (**a**) trapped-gas pores; (**b**) lack-of-fusion pores; (**c**) unmolten powder.

An incorrect choice of process parameters could lead to high levels of porosity within additively manufactured metal parts. Pores in additive manufacturing can be classified into two groups: trapped-gas pores and lack-of-fusion pores. Trapped-gas pores, as its name says, are the result of gas trapped inside the powder particles during gas atomization, or inside the printed part during the actual manufacturing. These pores have a rather spherical shape as can be seen in Figure 1a. Lack-of-fusion pores have, on the other hand, an irregular shape (see Figure 1b) and are much larger than trapped-gas pores. Lack-of-fusion pores appear when there is no complete adherence of the current melt to the surrounding part (when powder particles are only partially molten) [26]. These two types of pores possess intrinsically different characteristics, and therefore, they could affect/impact corrosion in different ways and/or to a different extent.

In general, close-to-the-surface pores can compromise the passivation properties of the native surface oxides, and therefore the pitting resistance of the material. This was demonstrated in previous studies for stainless steel [27,28], in which the researchers have shown that the level of porosity and the size of the pores play an important role in the resistance to pitting corrosion. However, no work has been conducted to date to systematically study the effect of porosity (dimension, type, and extent of porosity) on the corrosion behaviour of AM Al-based alloys. Therefore, more focus should be given to this issue in future studies. Moreover, pores might also play an important role in the susceptibility of the printed parts to stress corrosion cracking (SCC), since pores are intrinsically stress concentrators during mechanical loading. Once corrosive species reach the pores, occluded cell conditions could rapidly built-up and promote a premature failure of the printed part. This is a topic that needs more attention in the future.

As in the case of porosity, unmolten, or partially molten powder (see Figure 1c) results from incorrect choices of process parameters. Unmolten powder on the surface of the printed part can compromise the passive behaviour of the material by introducing defects in the native oxide film and greatly increases the surface roughness. There are currently several processes available (such as abrasive blast, shoot peening, electrochemical polishing, etc.) that are used to improve the surface finishing and reduce the roughness of AM metal parts [29,30]. These methods greatly affect the surface state and therefore, the surface energy, which influences the nucleation and growth of the native oxide film. This can, consequently, affect the barrier properties of the native oxide, and hence the passivity of the material.

**Figure 2:**
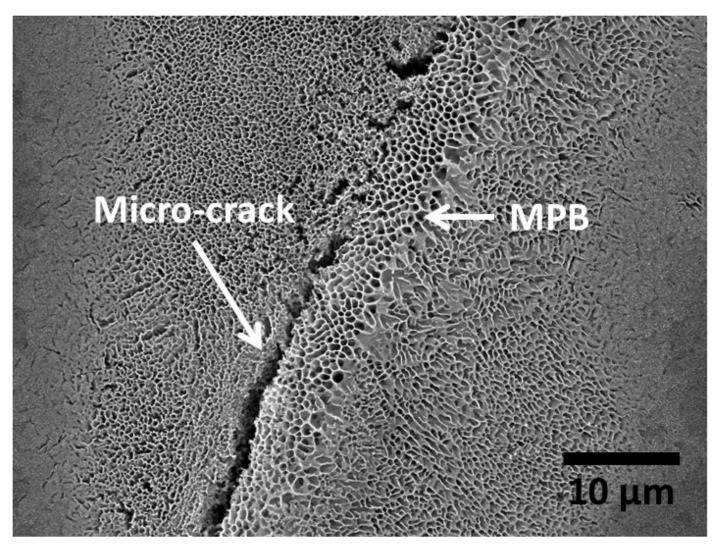
Secondary electron microscopy image of the surface of an as-built additive manufacturing (AM) Al-Si (AlSi10Mg) after immersion for 48 h in 0.1 M NaCl. The formation of micro-cracks in the heat affected zone next to the MPB can be seen. MPB—melt pool border.

Another intrinsic feature of additively manufactured metal parts that could compromise their physical integrity is the existence of residual stresses. Residual stress formation in metal additive manufacturing is caused by the high thermal gradients and cooling rates associated with these processes [31]. Particularly high tensile residual stresses are very typical near the surface [31]. These residual stresses can affect the materials’ susceptibility to stress corrosion cracking. Previous studies have shown the formation of micro-cracks in as-built AM Al-Si alloys after exposure to corrosive media (see Figure 2) [32,33]. The researchers associated this cracking with the combined effect of the local disruption of the Si network, the selective dissolution of the Al matrix around the disrupted zones, and the existence of residual stresses from the MAM process [33]. Additionally, surface residual stresses could, in general, influence the surface energy and therefore, the nucleation and growth of the native oxide film, affecting as a consequence the passivity and pitting resistance of the material. Nevertheless, no relevant work has been published so far on the study of the effect of residual stresses on the corrosion behaviour of additively manufactured metals.

## 3. Al-Si Alloys

### 3.1. Effect of Microstructure on Corrosion Behavior

**Figure 3:**
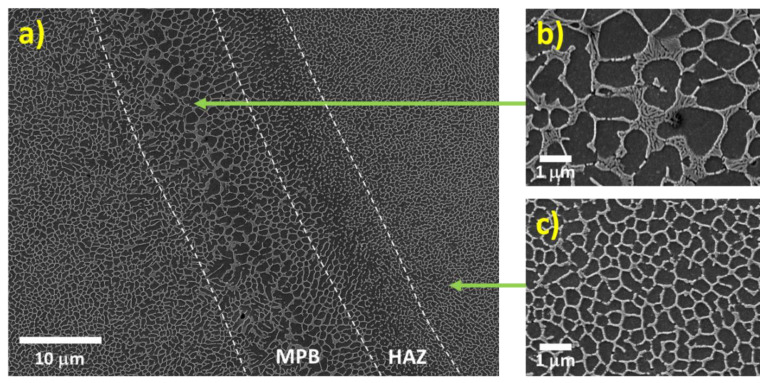
(**a**) Secondary electron image representing the microstructure of additively manufactured Al-Si alloys (AlSi10Mg). The surface parallel to the building platform is represented here. Similar features can be observed in the surface perpendicular to the building platform, but the shape of the cells is more elongated in that case. (**b**) Higher magnification image of a zone in the melt pool border. (**c**) Higher magnification image of a zone within the melt pool. MPB—melt pool border; HAZ—heat-affected zone. (Adapted from reference [43]).

Several studies characterizing the microstructure of as-built additively manufactured Al-Si alloys have been conducted in recent years [33,34,35,36,37,38,39,40,41,42,43]. As-built specimens exhibit a fine distribution of Si, forming a three-dimensional network that encloses the primary face-centred cubic α-Al in very small cells (see Figure 3). The size of these cells varies over the melt pool due to the thermal gradient created by the moving heat source. Finer cells are formed towards the middle of the melt pools (MP), while coarser cells are present in the melt pool borders (MPBs) [36]. A marked anisotropy has been described in past studies concerning the shape of these cells. These cells are known to present an approximately round shape in the plane parallel to the building platform (XY), whereas in the plane perpendicular to the building platform (XZ) more elongated cells have been observed [36,42]. A heat-affected zone (HAZ) located right outside the borders of the melt pools has also been identified (see Figure 3). This HAZ, in which the silicon network is partly broken and discontinuous, has been associated with overheating of the underlying layer during the scanning of a newly deposited layer [36].

A great number of works have already shown that the corrosion behaviour of additively manufactured Al-Si alloys is greatly influenced by this special and unique microstructure created during the additive manufacturing process.

#### 3.1.1. Influence of the Melt Pool Borders on Corrosion

**Figure 4:**
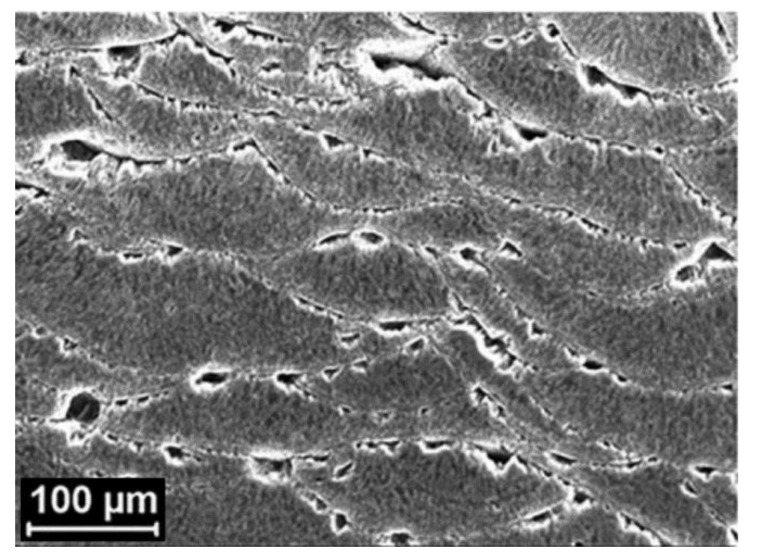
Secondary electron microscopy image of the surface of an as-built AM Al-Si (AlSi10Mg) corroded in NaCl solution. (Adapted from reference [47]).

Among the different details of the microstructural features of as-built AM Al-Si alloys, the borders of the melt pools have been pointed out to be a key element in the corrosion of these materials. Several studies have shown that after potentiodynamic polarization tests and/or immersion in NaCl solution a particularly pronounced selective corrosion of the α-Al cells in the melt pool borders is observed (see Figure 4) [32,33,44,45,46,47,48,49,50,51,52,53,54,55,56,57]. This selective corrosion attack along the borders of the melt pools has been reported for polished and ground specimens [32,33,44,45,46,47,48,49,50,51,52,53,54,55,56], but also for as-built materials presenting a low surface roughness [57], for which the microstructural features are believed to control the electrochemical performance of the material.

**Figure 5:**
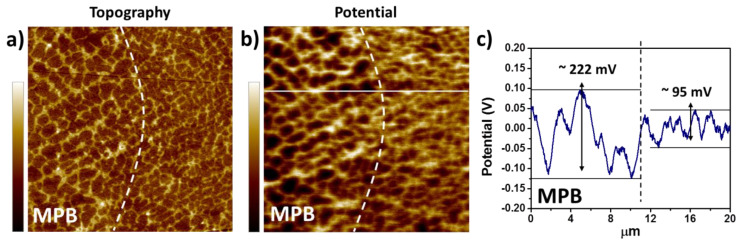
(**a**) Topography and (**b**) surface potential map (obtained by scanning Kelvin probe force microscopy—SKPFM) of an area on the surface of a polished AM AlSi10Mg specimen in which a melt pool border is visible (delimited by the discontinuous line). Scan size: 20 × 20 µm^2^. Colour bar: (**a**) 30 nm range, (**b**) 210 mV range. (**c**) Surface potential profile of the line represented in (**b**). (Adapted from reference [32]).

The initiation and further propagation of the corrosion attack in the Al cells along the MPBs are caused by the higher driving force for micro-galvanic corrosion between the α-Al and the Si phase in these regions compared to other areas within the melt pools [32,45,54]. Due to the presence of a relatively larger microstructure at the MPBs (see Figure 5), a greater Volta-potential difference has been reported between the Al and the Si phase compared to regions within the MPs (Figure 5c). Nevertheless, Kubacki et al. [51] do not support the assumption of a galvanic couple between the Al and Si phase accelerating corrosion in simulated atmospheric conditions at the melt pool borders. The researchers believe that under these conditions the cathodic kinetics on the Si phase is not fast enough to support the active dissolution of Al. Instead, the selective/pronounced corrosion attack at the borders of the melt pools is attributed to the high discontinuities of the Si network around the MPBs (in the heat-affected zone).

The morphology of the corrosion attack in as-built AM Al-Si alloys has been described by several researchers to be rather superficial due to the existence of a considerably connected Si network that holds the corrosion from penetrating deeper into the material [32,33,51,54,58]. However, several works have found a large penetration of the corrosion attack along the melt pool borders [46,50,51,52,53,55]. Moreover, the formation of micro-cracks during corrosion has been reported in regions next to the MPBs, where heat-affected zones exist [32,33,54,55,59]. As mentioned above, Revilla et al. [33] associated the formation of these micro-cracks to the synergistic effect of the selective dissolution of the α-Al cells along the MPBs, next to which the disrupted heat-affected zones are found, and the existence of internal and superficial residual stresses from the MAM process. Rafiezad et al. [49] also confirmed through intergranular corrosion test an accelerated preferential attack combined with the formation of micro-cracks along the melt pool borders for specimens fabricated using recycled powder. Moreover, while conducting electrochemical tests in NaCl solution, Girelli et al. [59] found that the MPBs/HAZ represent preferential paths for exfoliation-like corrosion to occur.

**Figure 6:**
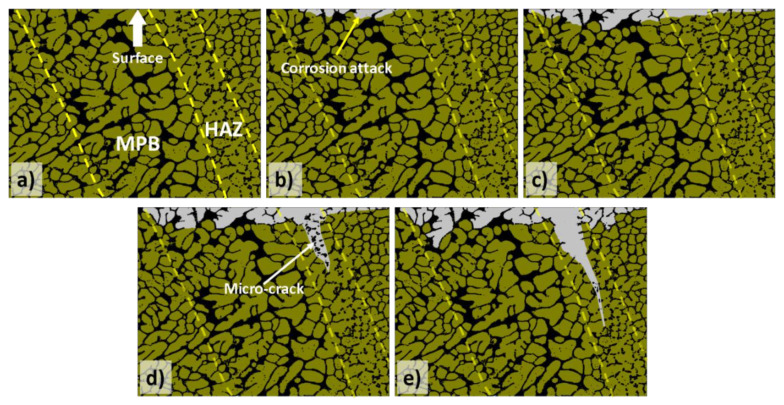
Schematic diagram representing the process of corrosion initiation and corrosion propagation for as-built additively manufactured Al-Si alloys. The illustrations portray the corrosion process seen from a cross-sectional perspective, with the top side of the images representing the surface exposed to the corrosive medium. The Al phase is represented with green, while black portrays the Si phase. A melt pool border (MPB), where a coarser microstructure is seen, and a heat-affected zone (HAZ), characterized by discontinuities in the Si network, can be observed in the images (**a**). The corrosion attack initiates at the MPB, where there is a larger potential difference between the Al and the Si phase, and therefore, a larger driving force for galvanic corrosion (**b**). Because of the partial containment of the corrosion by the Si network, the attack spreads superficially to adjacent zones, reaching the HAZ (**c**). Due to the disruption of the Si network in the HAZ, combined with the existence of internal stresses from the MAM process, micro-cracks are formed along the HAZ (**d**). In a later stage of the corrosion, additional lateral spreading of the attack occurs, accompanied by further propagation of the crack and corrosive medium through the HAZ (**e**) [33].

The schematic in Figure 6 shows the proposed evolution of the corrosion attack in as-built AM Al-Si alloys [33]. Because of the greater Volta-potential difference between the α-Al and the Si phase at the melt pool borders (as measured by scanning Kelvin probe force microscopy SKPFM [32,45,54]—see Figure 5), a higher driving force for galvanic corrosion provokes the initiation of the corrosion attack in these regions. The corrosion spreads superficially to adjacent zones, including the neighbouring heat-affected zones. This corrosion is partially contained by the connected portion of the Si network. However, due to the disruption in the heat-affected zone, the corrosion can propagate further into the material along the MPBs.

#### 3.1.2. Anisotropy

As already mentioned, the special microstructure of additively manufactured Al-Si alloys is the result of the exceptionally high cooling rates and direction of the thermal gradients [36]. The particular directional distribution of thermal gradients during the fusion and solidification of the different layers of the material produces an intrinsically anisotropic microstructure [36,42]. The small α-Al cells surrounded by the Si network are rather rounded in the plane parallel to the building platform, while in the plane perpendicular to the building platform these cells have an elongated shape [36,42]. Additionally, in the surfaces parallel to the building platform (XY plane), elongated laser tracks are easily identified, while in the surfaces perpendicular to the building platform (XZ plane) a scale-like feature of melt pool borders is generally seen [32,33,44,45,46,47,48].

Concerning the influence of this anisotropy on the corrosion behaviour/resistance of these materials, various and contradictory results have been reported in the literature. For AlSi10Mg prepared by SLM, Cabrini et al. [48,50] concluded from potentiodynamic polarization experiments in diluted Harrison’s solution that the surface of the XZ plane presents a slightly lower pitting corrosion resistance than the XY plane. The researchers associated this behaviour with the higher density of melt pool borders found in the XZ plane compared to that of the XY plane. However, in a later work, the same researchers concluded through a statistical approach that the building direction does not significantly influence the corrosion resistance of the analysed surface [56]. Moreover, Revilla et al. [32,33,54] found no difference in the electrochemical behaviour of the different planes during potentiodynamic polarization tests in aerated NaCl solution.

On the other hand, Cabrini et al. [46] demonstrated in another work by conducting an intergranular corrosion test that the corrosion in as-built specimens propagates mainly along the MPBs. Therefore, the penetration depth of the corrosion attack is highly influenced by the anisotropy of the melt pools. A more penetrative corrosion attack was seen on the surface parallel to the building platform compared to the surface perpendicular to the building platform. This could demonstrate that even if similar electrochemical behaviour is seen during potentiodynamic polarization tests, great anisotropy can still exist on the morphology of the corrosion attack and corrosion penetration depth.

**Figure 7:**
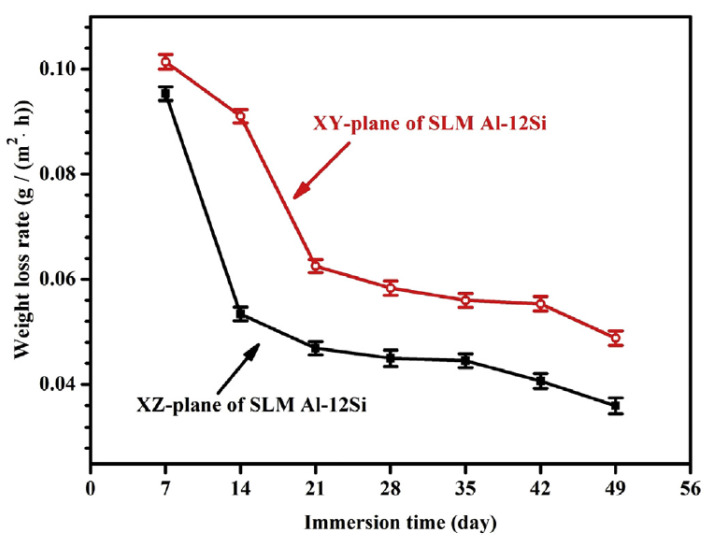
The weight loss rate for the different surface planes (parallel to the building platform—XY—and perpendicular to the building platform—XZ) of as-built and mechanically polished SLM AlSi12 after immersion in 3.5 wt.% NaCl solution at room temperature [42].

For SLM AlSi7Mg0.6 and AlSi12, no considerable difference was reported between the behaviours of the two different planes during potentiodynamic polarization tests in NaCl solution [33]. Nevertheless, a clear distinction in the corrosion resistance of the different planes was shown by Chen et al. [42] for SLM Al-Si12. The researchers presented experimental data (open circuit potential, potentiodynamic polarization, and electrochemical impedance spectroscopy measurements) supporting a better corrosion resistance of the XZ plane compared to the XY plane. This is the opposite trend as that reported for AlSi10Mg by Cabrini et al. [48,50]. Chen et al. [42] associated this behaviour with the depth of the Al/Si cells in each plane. The small and round cells were seen in the XY plane are deep since they are elongated in the perpendicular direction. According to the researchers, this could lead to the growth and even deposition of corrosion products, which could extrude and crack the Si shells, exposing the underlying Al substrate to further corrosion attack. On the other hand, the cells seen in the XZ plane are shallow, which could limit/prevent the deposition of corrosion products. Moreover, Chen et al. [42] reported a clear difference in the weight loss rate during corrosion in NaCl solution for the different planes. These results are shown in Figure 7. A reduced weight loss rate is seen for the XZ plane compared to the XY plane, possibly due to the same reason as that given for the difference in the electrochemical behaviour.

Due to the limited and contradictory results found in literature concerning the effect of the anisotropic microstructure on the corrosion behaviour, further research is needed to better understand this issue. It is also important to keep in mind that different alloys (even from the same alloy family) might display intrinsically different behaviours.

#### 3.1.3. Comparison with Conventional Alloys

While the microstructure of as-built AM Al-Si alloys presents a very fine distribution of alloying elements into a three-dimensional network, the traditional cast alloy is characterized by the presence of large Si crystals [32,44] and for the case of cast AlSi10Mg, other intermetallic particles such as AlFeSi and Mg_2_Si, among others, are also present [44]. These great differences in microstructure could result in differences in the corrosion behaviour of these materials. The corrosion resistance of AM Al-Si alloys has also been compared to that of conventionally manufactured alloys [32,44,51,59,60,61,62,63]. The results obtained for AlSi10Mg and AlSi12 are somewhat varied and in some cases contradictory.

##### AlSi10Mg

Table 1 summarizes, in a comparative way, the main results from potentiodynamic polarization tests obtained for AlSi10Mg. While some works report a similar corrosion current density for cast and AM AlSi10Mg [32,51,61], others claim a higher value for the cast alloy compared to the additively manufactured specimen [44,59,60]. Besides, while some studies show a similar value of corrosion potential for cast and AM AlSi10Mg [32,61], others report a nobler value of corrosion potential for AM AlSi10Mg in comparison with its cast counterpart [44,59,60]. On the contrary, Kubacki et al. [51] measured a lower value of corrosion potential for the additively manufactured material compared to the cast alloy. Nevertheless, the AM material presented a much higher value of pitting potential and a much wider passive region than the cast alloy [51].

While the corrosion attack in AM AlSi10Mg is mainly localized along the melt pool borders [32,33,44,45,46,47,48,49,50,51,52,53,54,55,56,57], the corrosion of the cast alloy is characterized by a severe localized corrosion of the α-Al matrix in the periphery of the large Si precipitates, as well as around the Mg_2_Si and Fe-containing intermetallics [32,44,51]. In general, several researchers claim that the corrosion resistance of additively manufactured AlSi10Mg is higher than that of the cast alloy of approximately the same chemical composition [44,60,61]. Girelli et al. [59] and Leon et al. [61] found a slightly lower mass loss during corrosion in aerated 3.5 wt.% NaCl for the AM specimen compared to the cast alloy. However, Kubacki et al. [51] concluded that while both the cast and AM AlSi10Mg presented similar surface damage and median corrosion depth after immersion in a modified G85-A2 (cyclic acidified salt fog/spray test) cycle, the additively manufactured material presented a greater maximum damage depth compared to the cast alloy.

##### AlSi12

The AlSi12 alloy has been, in general, less studied than AlSi10Mg. Only a few works have compared the corrosion resistance of the additively manufactured specimens with that of the conventional cast alloy [62,63]. Yang et al. [63] reported a higher corrosion resistance for the AM material compared to the cast alloy. Their experimental data in NaCl solution showed not only a better electrochemical performance during open circuit potential and potentiodynamic polarization tests for the AM AlSi12 sample than for the cast alloy, but also a much lower weight loss during immersion in the same solution for the AM sample compared to the cast material [63]. On the other hand, other researchers reported that the cast and the AM AlSi12 alloy display similar corrosion behaviour in the HNO_3_ solution [62]. Weight loss measurements after immersion in 0.1 M HNO_3_ showed an almost perfect overlap between the curves of the cast and additively manufactured AlSi12 alloy [62].

### 3.2. Effect of Heat Treatment on Microstructure and Corrosion

As mentioned above, the unique processing conditions during MAM cause rapid solidification of melt pools due to the extremely high cooling rates reached (~10^5^ K/s) [39,58]. This leads to a very fine cellular microstructure far from equilibrium, consisting of a primary Al-rich phase [39,64] supersaturated in Si (up to 11 wt.%) [52] with residual Si segregated at the cellular boundaries [39]. The extremely fine Si network gives a large total interfacial energy, resulting in a high driving force for Si coarsening [38]. As thermal gradients and grain growth rates decrease towards the melt pool borders, coarser cellular structures can be found there [36]. In addition, the fast cooling rates can introduce residual thermal stresses that can lead to dimensional inaccuracy and distortion [55]. A variety of heat treatments can be performed to act on these factors. Due to the presence of Mg in most Al-Si alloys, artificial aging (150–180 °C) [65] can be performed to induce age hardening through Mg_2_Si precipitation [34,52,55]. The increase in the solid solubility of Si in Al due to the rapid solidification can enhance the effect of solution hardening and strengthening [66]. Partial annealing treatments can be performed to release residual stress [50,52,53,54,55,58,67,68]. Alternatively, the building platform can also be heated during manufacturing [34,52].

Fiocchi et al. [69] performed Differential Scanning Calorimetry (DSC) experiments to determine the phase transformations occurring in an AlSi10Mg alloy prepared by selective laser melting. Several heating rates (2, 5, 10, 20, and 30 °C/min) were applied for a temperature range of 0 to 500 °C. Two exothermic transformations were identified for the SLM material. The first peak was found to be common to SLM material, unmolten powder, and cast alloy of similar composition. It was determined to result from the alloying elements and was identified as Mg_2_Si precipitation. The second peak was found to be unique to the SLM material. It was ascribed to the rupture and spheroidization of the Si network, which is characteristic of SLM parts. The isothermal endpoint temperatures of these transformations were calculated using second-degree polynomial regressions, resulting in 263 °C for Mg_2_Si precipitation and 294 °C for Si rupture and spheroidization [69]. Similar DSC experiments on SLM AlSi10Mg alloy were performed by Rafieazad et al. [55] for a temperature range of 0 to 550 °C. They determined the peak values for the two exothermal transformations at several heating rates (2, 5, 10, and 20 °C/min) and calculated the isothermal temperatures using second-degree polynomial regressions. These were determined to be 232.9 and 273.2 °C. Similar to the work of Fiocchi et al. [69], these were interpreted as being respectively the Mg_2_Si β phase precipitation and the Si phase precipitation via solid-state diffusion [55].

#### 3.2.1. Effect on Microstructure

##### Stress Release Heat Treatments

Several studies have focused on the effect of stress-relieving heat treatments on the microstructure. Cabrini et al. [52] studied the effect of stress-release at 200, 300, 400, and 500 °C for 2 h. Similarly, Rubben et al. [54] investigated the effect of stress-release at 250 and 300 °C for 2 h, while Rafieazad et al. [55] investigated heat treatments at 200, 300, and 350 °C for 3 h. Other papers discuss annealing with similar conditions [68] but without the expressed aim of stress release.

**Figure 8:**
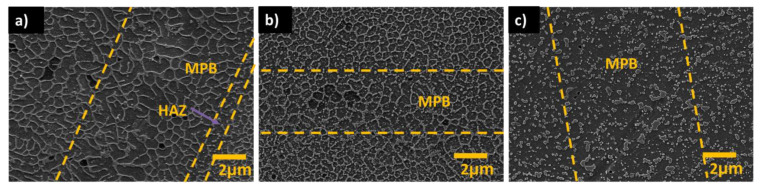
Secondary electron image showing the microstructure of (**a**) an as-built AM Al-Si sample (AlSi10Mg), as well as two stress-released specimens: one at 250 °C for 2 h (**b**), and another at 300 °C for 2 h (**c**). MPB refers to the melt pool border, while HAZ stands for the heat-affected zone. (Adapted from reference [54]).

Cabrini et al. [52] observed that stress release heat treatments up to 300 °C for 2 h do not cause a significant change in the characteristic melt pool macrostructure. It should be noted however that although melt pool tracks are retained, significant microstructure alteration can still occur. For the heat treatment of 200 °C for 3 h, no microstructure change was noticed [55]. This is attributed to the temperature being significantly lower than the minimum required for Si interdiffusion in Al [55]. After a stress release at 250 °C for 2 h, a limited breakup of the Si network was observed [54] (see Figure 8b). The limited nature of this breakup can be explained by considering that the temperature of stress release was below the peak temperature value of Si network coarsening [55], resulting in slow kinetics. More significant changes occurred after stress release at 300 °C for 2 h. The morphology of the Si phase changed completely, going from a continuous silicon network towards separate Si precipitates (see Figure 8c) [52,54,68]. Despite this, larger microstructures were still observed at MPBs. As a result, the melt pool macrostructure was retained. After stress release at 400 °C for 2 h, the MPBs became harder to distinguish [52]. Rafieazad et al. [55] reported a similar diminishing of MPBs after 3 h at 350 °C. In both cases, an α-Al matrix with rounded silicon particles was revealed at higher magnification [52,55,68]. When the temperature was increased to 500 °C, the characteristic melt pool macrostructure completely disappeared. Silicon particle coalescence occurred, leading to much larger silicon particles [52].

##### Heating of Building Platform

Heating the building platform during manufacturing can be used as an alternative to the stress release heat treatment. Residual stresses can be reduced by increasing the building platform temperature without affecting the mechanical strength [52]. Cabrini et al. [52] studied the effect of two different building platform temperatures, 35 and 100 °C. The temperature change did not cause a noticeable change in the macrostructure as both show similar melt pools and heat-affected zones. At higher magnification, some small changes were noticed in the melt pools. Limited formation of the eutectic-like phase by silicon particles was more visible for specimens built at 100 °C. The small changes in microstructure explain the lack of change in mechanical strength. A higher building temperature of 300 °C was investigated by Brandl et al. [34]. No significant change in macrostructure was obtained. It should be noted that the used magnification was not high enough to be able to observe variations in the cellular network.

##### Solution Treatment—T6

**Figure 9:**
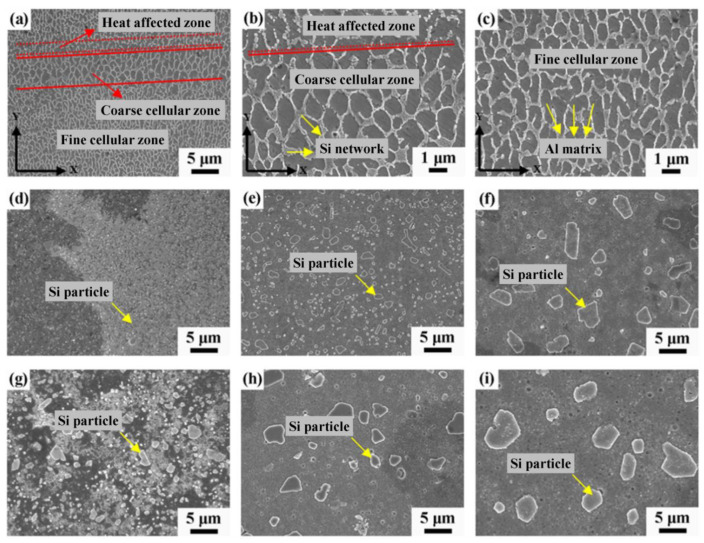
Secondary electron micrographs showing the microstructure of as-built and heat-treated AM AlSi10Mg. (**a**–**c**) Represent the microstructure of the as-built samples in different zones and at different magnifications. (**d**–**i**) Represent the microstructure of the samples after different heat treatments: (**d**) 450 °C for 2 h; (**e**) 500 °C for 2 h; (**f**) 550 °C for 2 h; (**g**) 450 °C for 2 h + 180 °C for 12 h; (**h**) 500 °C for 2 h + 180 °C for 12 h; (**i**) 550 °C for 2 h + 180 °C for 12 h. (Adapted from reference [38]).

Several researchers [34,38,65] have focused on the T6 heat treatment, consisting of a solution treatment (450–550 °C) [38,58] followed by quenching and artificial aging (150–180 °C) [65]. Both Li et al. [38] and Gu et al. [58] solution treated SLM produced AlSi10Mg material at 450, 500, and 550 °C for 2 h, followed by water quenching. In both cases, results showed that the microstructure became coarser with increasing solution temperatures (see Figure 9). After solution treatment at 450 °C for 2 h (see Figure 9d), Si precipitates were small (<1 µm) and uniformly distributed in the Al matrix. When the temperature was further increased to 500 and 550 °C (see Figure 9e,f), particle coalescence and Oswald ripening occurred, resulting in a further increase in particle size (2–4 µm) [38]. The volume fraction of Si increased, proving that Si precipitates during heat treatment [58]. In the work of Li et al. [38], half of the specimens additionally underwent artificial aging at 180 °C for 12 h after the solution treatment. Further coarsening occurred with the addition of the artificial aging procedure (see Figure 9g–i), despite the temperature being seemingly too low to induce Si coarsening [38,55]. This is in contrast to the work of Aboulkhair et al. [65], where a solution treatment at 520 °C was applied for 4 h. The addition of 6 h of artificial aging at 160 °C did not seem to alter particle size or density.

##### Artificial Aging Heat Treatments

In most studies, artificial aging (AA) heat treatments are performed after a solution treatment (e.g., T6 heat treatment). Kempen et al. [23] noted that this is not ideal for SLM parts as it undoes the fine microstructure giving the superior mechanical properties. The effect of AA heat treatment on the microstructure of as produced SLM material was studied by Rubben et al. [54]. No significant microstructure evolution was observed compared to the untreated specimens. An explanation for this can be found in the works of Fiocchi et al. [69] and Rafieazad et al. [55]. The temperature of the heat treatment is significantly below the peak temperature (273 °C [55]) of precipitation, coarsening, and spheroidization of Si particles.

#### 3.2.2. Effect on Corrosion

##### Stress Release Heat Treatments

Several studies have focused on the effect of stress-relieving heat treatments on corrosion behaviour. Cabrini et al. [52] studied the effect of stress release for 2 h at 200, 300, 400, and 500 °C. They performed intergranular corrosion tests in 30 g/L NaCl + 10 mL/L of HCl at room temperature for 24 h. Another study by Rubben et al. [54] focused on the effect of stress release for 2 h at 250 and 300 °C. To achieve this, they performed open circuit potential, potentiodynamic polarization, and immersion experiments in 0.1 M NaCl.

On untreated specimens, selective corrosion attacks occur at the melt pool borders [52,54]. Galvanic coupling between α-Al and Si causes the dissolution of Al (as already explained above). Selective corrosion attacks at MPBs were still detected after performing stress release up to 300 °C for 2 h [52,54]. This is despite significant microstructure evolution, with the microstructure changing from a continuous silicon network to separate Si precipitates, with larger precipitates formed at the MPBs. Revilla et al. [32] and Rubben et al. [54] performed SKPFM measurements on specimens before [32,54] and after stress release at 300 °C for 2 h [54]. For both cases, measurements showed Volta potential differences between the primary aluminium and the more noble Si, with larger Volta potential differences at the melt pool borders compared to the interior of the melt pools. This indicated that the larger microstructure found at the MPBs is more prone to galvanic corrosion, explaining the selective attack at the MPBs before and after heat treatment.

**Figure 10:**
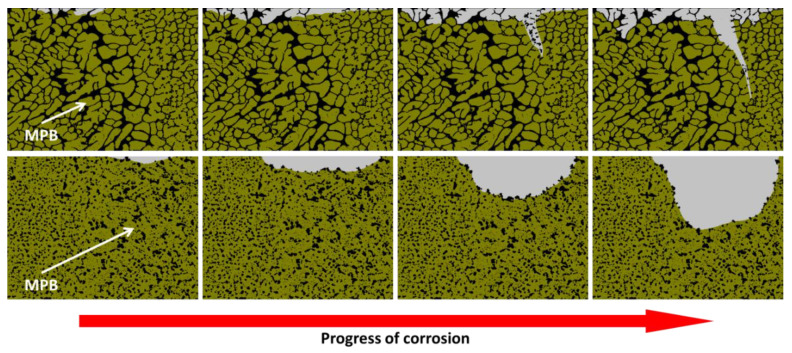
Schematic diagram representing the evolution of the corrosion attack for two separate cases: (**top**) When a connected Si network is present, as is the case for as-built AM Al-Si specimens, and (**bottom**) when the Si is broken up in separate precipitates, as is the case for heat-treated AM Al-Si parts at relatively high temperatures (~300 °C or higher). The illustrations portray the corrosion process seen from a cross-sectional perspective, with the top side of the images representing the surface exposed to the corrosive medium. The Al phase is represented with green, while black portrays the Si phase. For as-built as well as heat-treated specimens, the corrosion initiates at the melt pool borders (MPB), due to the higher driving force for galvanic corrosion in these regions. For as-built specimens corrosion spreads superficially, accompanied by the formation of micro-cracks along the heat-affected zones. On the other hand, a dip and relatively wide penetration of the corrosion attack characterizes heat-treated specimens due to the presence of separate Si precipitates. (Adapted from reference [54]).

While preferential corrosion at MPBs was noticed for these types of specimens, Rubben et al. [54] illustrated that the underlying mechanism may change depending on the temperature of stress release. This was linked to a corresponding change in microstructure (as explained in the previous section). When the Si network is still intact (which is the case for untreated specimens, and heat-treated at 200 °C), the corrosion is mostly superficial with microcrack formation at the heat-affected zone next to the MPBs. This allows corrosion to locally spread deeper in the material. When the Si network has broken up (for instance after stress release at 300 °C for 2 h), the spread of corrosion is no longer stopped by the presence of an Si network, leading to a more broadly penetrating attack around MPBs. This behaviour is summarized in Figure 10. Depending on how discontinuous the silicon network is, both mechanisms may be active at the same time [54].

After stress release at 400 or 500 °C for 2 h, no more selective attacks at MPBs were detected after intergranular corrosion tests [52]. Instead, more general corrosion morphologies were noticed. This was linked to a significant modification of the microstructure, showing an α-Al matrix and rounded precipitates of Si. This leads to the disappearance of the characteristic melt pool macrostructure. As there is no longer a larger microstructure at the MPBs, no preferential attack occurs in those cases.

##### Heating of Building Platform

Intergranular corrosion tests were performed by Cabrini et al. [52] on specimens fabricated with a building platform temperature of either 35 or 100 °C. Specimens were immersed for 24 h in 30 g/L NaCl + 10 mL/L of HCl at room temperature. The corrosion morphology showed a superficial attack, with a deeper attack for specimens built at 35 °C. This change can be attributed to either the small change in microstructure (see the previous section) or to the reduction of thermal stresses. The latter is supported by the works of Revilla et al. [32,33,54], where it was postulated that the presence of residual stresses combined with the exposure of the silicon network after corrosion might lead to the development of micro-cracks, allowing the spread of corrosion further in-depth.

##### Solution Treatment—T6

Some research has been performed on the effect of solution heat treatments on the corrosion behaviour of SLM AlSi10Mg. In one study, Gu et al. [68] solution treated SLM produced AlSi10Mg material at 300 and 400 °C for 2 h under an argon atmosphere, followed by water quenching. In another study by Gu et al. [58], SLM produced AlSi10Mg material was solution treated at 450, 500, and 550 °C for 2 h under an argon atmosphere, followed by water quenching. In both studies, experiments were conducted in 3.5 wt.% NaCl solution.

In untreated specimens, the continuous Si network impedes contact between the underlying metal matrix and the electrolyte, restricting the transfer of Al^3+^ ions towards the electrolyte [58]. After heat treatment, the continuous Si network devolved into separate Si precipitates (see the previous section). Higher temperature heat treatments, coinciding with the growth of the Si particles, resulted in higher corrosion current densities and lower corrosion potentials, film resistances, and charge transfer resistances [58,68]. This was attributed to the change in the morphology of the Si phase. Larger separate Si precipitates restrict the formation of a compact oxide layer [58,68] while Si precipitating from the Al matrix leads to an increasing cathode area and a more active matrix [58]. Further explanations can be found by looking at the work of Revilla et al. [32]. SKPFM measurements showed that the Volta potential difference between the Si phase and the matrix increases with the size of the particles due to the influence of the surrounding matrix. As the Si particles grow in size with increasing temperature, a higher driving force for galvanic corrosion is obtained.

##### Artificial Aging Heat Treatments

Rubben et al. [54] studied the effect of AA on the corrosion properties of as produced SLM specimens. Open circuit potential (OCP), linear sweep voltammetry (LSV), and immersion experiments in 0.1 M NaCl showed a similar behaviour, resulting from the lack of significant changes in microstructure compared to the untreated specimens (see the previous section). This shows that performing the artificial aging without prior solution treatment is not only critical for retaining the mechanical properties [36] but also for retaining the corrosion resistance of SLM material.

### 3.3. Effect of Si Content on Microstructure and Corrosion

Even though several studies have been dedicated to investigating the corrosion behaviour of AM AlSi10Mg [32,33,44,45,46,47,48,49,50,51,52,53,54,55,56,57,58,59,60,61,68,70], and a few other works have studied the corrosion performance of AlSi12 alloy [33,42,62,63], very limited research explores the influence of Si on the microstructure and corrosion behaviour of these materials. A recent comparative study carried out on polished as-built AlSi7Mg0.6, AlSi10Mg, and AlSi12 demonstrated that the general appearance concerning the structure of melt pools for all of these additively manufactured specimens was approximately the same [33]. All the specimens, independently on the amount of Si in the alloy, presented a fine cellular structure within the melt pools, while at the borders coarser cells were detected. Additionally, heat-affected zones, in which idiomorphic Si particles and a relatively more discontinuous Si network are present, were identified next to the MPBs for all of these specimens. Nevertheless, higher resolution SEM analysis revealed that even though the size of the Al cells was independent of the amount of Si in the alloy, the connectivity of the Si network was highly affected by the Si content. A much thinner and partially broken Si network was observed for the as-built AlSi7Mg0.6 specimen, while a thicker and much more connected Si network was seen in the as-built AlSi12 sample. By increasing the amount of Si, a greater level of connectivity is observed in the silicon network of the AM specimens. Similarly, a relatively higher level of connectivity was seen in the silicon network within the HAZ for higher amounts of Si [33].

The corrosion studies on these three AM specimens revealed that, even though the corrosion attacks seemed rather superficial for all the samples, the corrosion resistance in NaCl solution is slightly influenced by the specific alloying content: materials with higher amounts of Si (and lower Mg content) showed higher resistance against corrosion [33]. The corrosion resistance of the materials showed the following relationship: AlSi12 > AlSi10Mg > AlSi7Mg0.6. The relatively low resistance against corrosion for the AlSi7Mg0.6 specimen could be due to the higher amount of Mg present in this material compared to the others, since the presence of Mg in Al-Si alloys has repeatedly been shown to greatly influence their corrosion behaviour through the formation of Mg_2_Si precipitates [71,72,73]. Furthermore, earlier research has shown that the corrosion resistance of Al-Si alloys decreases by increasing the content of Mg [73]. Nevertheless, it is important to notice that (to the best of our knowledge) the actual formation of Mg_2_Si particles in additively manufactured Al-Si alloys has not yet been confirmed, due possibly to the extremely high cooling and solidification rates resulting in a very fine (out-of-equilibrium) distribution of alloying elements with almost no time for the formation of the usual precipitates. We believe that the possible formation of Mg- and Fe-containing precipitates in these materials should be more systematically studied in future works.

Moreover, due to the relatively high connectivity of the Si network in the as-built AM AlSi12 material, no micro-cracks were formed after corrosion; while for the case of AlSi7Mg0.6 and AlSi10Mg several micro-cracks were seen at heat-affected-zones next to the MPBs [33]. This selective cracking seems to be caused by the combination of several factors: the relatively larger disruption of the Si network in the HAZ, the selective dissolution of the Al matrix around the MPBs/HAZ due to the presence of a corrosive medium, and the existence of residual internal stresses from the MAM process [33].

Cabrini et al. also conducted a comparative study using AlSi7Mg0.6 and AlSi10Mg to investigate the combined effect of Si content and heat treatment on the susceptibility of these materials to corrosion attack [46]. They demonstrated that in the case of as-built (untreated) specimens, higher Si content in the material resulted in a lower penetration depth of the corrosion attack. As mentioned above, this could be due to the higher level of connectivity achieved in the Si network for higher amounts of Si in the alloy [33]. On the other hand, after heat treatments, the Si network (partially) breaks into isolated Si particles, resulting in greater penetration depth of the corrosion attacks for samples with the highest Si content. For higher amounts of Si, larger Si particles will be formed after heat treatment. These isolated Si particles present a more cathodic potential than the Al matrix, being the main cause for the localized corrosion in these materials (due to galvanic coupling effect). For larger Si content and Si particles formed after heat treatment, larger will be the extent of this corrosion attack, which will then not be contained/stopped by the (partially) broken Si network.

### 3.4. Effect of Surface Roughness on Corrosion

In order to study the effect of surface roughness on the corrosion behaviour of additively manufactured Al-Si alloys, several works compare the corrosion performance of as-built AM specimens with that of polished or ground samples [44,50,53,60,70]. In most of these cases, it was shown that the corrosion resistance in NaCl solution [44,50,53,60], as well as the low cycle corrosion fatigue life span [70], is improved after mechanical polishing. The reduced corrosion resistance of as-produced SLM samples was ascribed to the excessive number of cavities and other surface defects generated during the SLM process at the external sample surface. These surface defects will induce localized corrosion in the form of pits, which could then be considered as crack initiation sites. The stimulated crack initiation and propagation of the unpolished samples thus resulted in a relatively accelerated corrosion fatigue failure [70]. Nevertheless, while studying the effect of surface finishing on the corrosion properties of AlSi10Mg prepared by direct laser sintering, Fathi et al. [60] concluded that during the initial stage of immersion in NaCl solution, SLS-prepared samples had a higher corrosion resistance than ground specimens. Ground specimens were characterised by a high selective attack mainly at the heat-affected zones next to the MPBs, while only a minor attack on the surface of as produced samples was seen. The authors believe that this is caused by a less protective passive film on the ground samples. However, for long immersion times, a change in corrosion behaviour was seen. It was reported that the ground SLS samples now had the highest corrosion resistance. This might indicate that during the initial stage of immersion in a corrosive medium, the internal microstructural features (such as MPBs), which are more pronounced for polished/ground specimens, play a more dominant role in the corrosion process.

An improved surface quality of AM samples is, however, not always possible to obtain by post-printing operations, like mechanical polishing/grinding due to the complex shape of the final printed parts. Therefore, methods such as shot peening and sandblasting (among others) are sometimes applied in order to improve the surface quality of the as-built parts. Some works have been dedicated to studying the effect of such methods on the corrosion behaviour of these materials. The results demonstrate that shot-peened and sandblasted materials present a slightly higher resistance against corrosion than as-built parts due to the reduction of surface roughness and superficial defects [50,60]. However, a much better corrosion performance is obtained always for the polished or ground samples. Fathi et al. [60] propose that it is of the utmost importance to perform a post-grinding operation, in order to substantially improve the corrosion properties of the printed parts. Cabrini et al. [48] further showed an improvement of the corrosion resistance of as-produced parts by bright dipping in a phosphoric/nitric acid bath, attributed to the removal of the oxide film formed during printing. Moreover, they noticed that the corrosion resistance worsened after a bright dipping of mechanically polished parts, caused by the silicon enrichment at the surface during etching.

Furthermore, the surface roughness can also be improved by changing the printing parameters. Calignano et al. [74] studied the effect of different printing parameters on the surface roughness of AlSi10Mg DMLS produced samples. They found that the scanning speed had the greatest influence on the surface roughness, followed by the hatching distance. Fathi et al. [57] studied the corrosion properties of as-produced SLS samples with improved surface quality. The reduced surface roughness was obtained by adjusting the beam offset and reducing the scanning speed and hatching distance, as was suggested by Calignano et al. [74]. They reported that by changing the printing parameters, like the hatch distance, not only does the surface quality change for the better, but the degree of overlap between melt pools changes as well. Furthermore, this study showed that by reducing the hatch distance a material with periodically large and small melt pools is obtained. This overlap between melt pools was shown to play an important role in the solidification behaviour: for higher overlap, the higher the solidification rate will be. The authors claimed that in those cases not the surface roughness, but the resulting microstructure has the largest effect on the corrosion properties of the as-produced parts. Moreover, the material with the finest microstructure, originating from the higher degree of melt pool overlap, showed the best corrosion properties. This was attributed to the fact that the coarser the microstructures of the Al dendrites and the Si particles are, the higher the galvanic coupling and galvanic corrosion [57].

### 3.5. Corrosion Protection

Even though several studies have demonstrated that the special conditions during MAM have a great influence on the microstructure and corrosion behaviour of these materials, not many works have been dedicated to investigating the influence of their special microstructure on the mechanisms of corrosion protection. To date, only a few studies explore the impact of microstructure, heat treatments, Si content, and defects on the anodizing behaviour of Al–Si alloys prepared by selective laser melting. Anodizing of aluminium and aluminium alloys is done to improve their corrosion protection. Galvanostatic anodizing of SLM produced Al-Si alloys in H_2_SO_4_ electrolyte was studied by Revilla et al. [41,43,67,75]. They concluded that it is possible to anodize SLM produced Al–Si alloys. However, it was shown that the characteristic microstructure of SLM produced parts will have a significant impact on the voltage-time response and on the formed anodic oxide film. Furthermore, they showed a rather significantly different anodizing behaviour when compared to the cast alloy of similar chemical composition. The voltage-time response of the cast alloy shows the typical steady-state growth regime, while the AM material, on the other hand, showed a continued increase in voltage until eventually a steady-state was reached. Due to the fine distribution and high connectivity of the Si network in as-built AM Al-Si alloys, the moving oxide front will be obstructed to a larger extent resulting in the formation of a thinner oxide film. Moreover, a significantly lower oxide growth rate was seen for the SLM alloy compared to the cast alloy. This lower oxide growth rate was attributed to a larger fraction of the anodic charge consumed by Si oxidation in the SLM alloy. The effect of the Si distribution on the anodizing behaviour of Al-Si alloys was also studied [43]. It was shown that the melt pool borders in SLM produced parts have a eutectic Al-Si structure with alternating lamellae consisting of Al and Si. This lamellae structure was attributed to the lower cooling rate in these melt pool borders compared to the centre of the melt pools. Furthermore, it was reported that in these melt pool borders the Al content was slightly lower compared to the average value in the whole sample, and the Si content slightly higher [43]. This slightly higher Si content and eutectic Al-Si lamellar structure in the melt pool borders was suggested to cause the relatively thinner oxide film formed at these melt pool borders. An XPS analysis reported that in the anodic film of the cast alloy only a superficial layer of the Si precipitates is oxidized. However, in the SLM samples, most of the Si in the anodic film is oxidized; this was suggested to cause a severe reduction of the anodizing efficiency compared to the cast alloy. It was shown that the anodic film formed on the cast alloy has a much higher roughness than the anodic film formed on the AM specimen. This roughness difference was attributed to the difference in aluminium cell size between the cast alloy and the SLM alloy [43].

Moreover, a great anisotropy was also seen during the galvanostatic anodizing of as-built AM Al-Si samples [41,75]. The voltage–time response curves of the AM material showed a different response depending on the orientation of the surface that was anodized. The voltage–time response of a surface parallel to the building platform (XY surface) was shown to give a higher steady-state potential value than that of a surface with an orientation perpendicular to the building platform (XZ surface). This asymmetric anodizing behaviour was found to be independent of the Si content in the alloy. The authors proposed that this asymmetry could be related to the difference in the size of aluminium cells in the different planes [41], or differences in the density of melt pool borders encountered by the anodizing front [75].

A unique pore structure was seen for the SLM alloys. A branched-like pore structure was seen throughout the whole anodic film. This pore structure was attributed to the fine distribution of the silicon phase in an almost continuous network encapsulating the aluminium in small cells [41]. Revilla et al. [75] further reported that the Si content in the alloy has a significant effect on the pore structure of the anodic film, i.e., the higher the Si content the higher the voltage response will be. As a consequence of the higher voltage response, wider pores with a greater inter-pore distance are obtained. Furthermore, the pore density is shown to decrease with Si content.

Rubben et al. [67] studied the effect of several heat treatments on the anodizing behaviour of SLM produced AlSi10Mg. It was shown that anodizing behaviour depends strongly on the morphology of the Si phase, which can be highly affected by the heat treatment applied. For the non-heat treated and the artificially aged (at 170 °C for 6 h) samples, the Si phase consists out of a rather continuous network, consequently, most of the Si encountered by the anodizing front will be oxidized. For the stress released heat-treated samples (at 250 and 300 °C for 2 h), the Si network is broken up into separated Si precipitates [67]. As a consequence, only a fraction of the Si phase will be oxidized. It was shown that the difference in Si phase morphology can give rise to significantly different anodizing behaviour. Furthermore, they reported that voltage–time response during anodizing varied strongly depending on the heat treatment applied. The steady-state anodizing potential was reported to increase with the anodized Si fractions [67]. Additionally, the anodizing efficiency was shown to decrease with a higher anodized Si fraction.

Finally, Revilla et al. [75] investigated the influence of internal pores, resulting from the MAM process, on anodizing behaviour. They showed that when the moving oxide front encounters such a pore, the sides of the pores get anodized. Furthermore, the sides of the anodized internal pores showed cracks, probably caused by the volume expansion of anodic layers advancing in opposite directions [75]. Even though the cracks observed were relatively small (1–2 µm), they could compromise the physical integrity of the final piece. Additionally, these cracks could facilitate the access of corrosive media to the metal matrix.

Other studies are needed in the future to further understand the anodizing behaviour of these materials under other anodizing conditions such as: potentiostatic anodizing regime, different electrolytes, and study the effect of surface roughness. Moreover, the effect of microstructure and microstructural defects on other surface treatments aimed at the protection of the materials against corrosion should also be investigated.

## 4. Other Al Alloys

Besides the Al-Si alloys discussed in previous sections, only very few other additively manufactured Al-based alloys have been considered so far for corrosion studies. The only other cases are, to the best of our knowledge, AA2024 and AA7075 [76,77]. For AA2024, the main alloying agent added is Cu, while for AA7075 the primary alloying element is Zn. The specifications concerning the chemical composition of these alloys are given in Table 2. AA2024 is a heat-treatable alloy widely used in aircraft structures due to its high strength to weight ratio, as well as good fatigue resistance. AA7075 has high specific strength, low density, and good thermal properties, for which it is widely used in transport applications, including marine, automotive, and aviation, as well as in-mold tool manufacturing.

Previous work on polished wrought and additively manufactured AA2024 revealed that due to the special conditions associated with the selective laser melting process (i.e., highly localized melting and subsequent rapid solidification), the microstructure of AM AA2024 was characterized by a refined particle size [76]. The traditional micrometre-sized constituent particles and the S-phase Al_2_CuMg present in wrought AA2024-T3 were instead replaced by nm-sized particles, which were mainly determined to be θ-phase Al_2_Cu. This highly refined microstructure was shown to greatly impact the corrosion behaviour of this material according to results obtained in polished samples [76]. While the anodic current was found to increase rapidly for potentials above the corrosion potential during anodic polarization of wrought AA2024-T3, the additively manufactured AA2024 materials presented a passive-like window. This could be due to the finer microstructure found in the additively manufactured material compared to the wrought specimen, which resulted in a thicker and more stable native oxide film; as well as the absence of S-phase precipitates in AM AA2024. It was also shown that the corrosion rate of the Al matrix in 0.01 M NaCl for the AM AA2024 material was about five times lower than that measured in wrought AA2024-T3. Moreover, the dissolution ratio between the alloying elements (Cu and Mg) and that of Al was about ten times higher for the AM AA2024 sample than for the wrought AA2024-T3. As the authors stated, the dissolution of these alloying elements at the early stages of corrosion is beneficial since it reduces the number of cathodic sites that would be detrimental for subsequent localized corrosion.

The relation between microstructure and corrosion behaviour has also been studied for AA7075 materials [77]. Gharbi et al. [77] demonstrated that while coarse (up to approximately 15 µm in size) second phase Al-Cu-Fe(-Si) particles exist in the traditional wrought AA7075-T6 material, finely distributed nm-sized Mg-Zn-Cu(-Al) (ν-phase) and Mg_2_Si (β-phase) characterize the microstructure of additively manufactured AA7075 prepared by SLM. This microstructure was also shown to vary significantly after solutionising and subsequent artificial ageing. The existing ν-phase in as-built specimens was dissolved after solutionising, while the subsequent aging resulted in the formation of MgZn_2_ precipitates. Concerning their corrosion behaviour, the authors demonstrated that, depending on the post-heat-treatment, AM AA7075 presented a higher corrosion resistance compared to wrought AA7075-T6 [77]. While as-built AM AA7075 and wrought AA7075-T6 presented a highly active behaviour during anodic polarization in NaCl solution, the heat-treated specimens presented a passive-like behaviour. The immersion of the samples in a NaCl solution revealed that pits formed in as-built AM AA7075 were notably smaller compared to those formed in wrought AA7075-T6, possibly due to the fine microstructural features and the absence of large second phase particles in the AM material compared to its wrought counterpart. Moreover, while pitting occurred predominantly along the melt pool borders in the case of polished as-built AM AA7075, pits were uniformly distributed on the Al matrix for the solutionised AM material. Due to the formation of MgZn_2_ precipitates in the solutionised aged samples, large pits were formed along grain boundaries [77].

To enhance the metallurgical state of the AA7075 alloy, and therefore, make it more suitable for processing by additive manufacturing, the minor addition of transition elements such as Sc, Zr, Ti, B, Fe, and Ni has been explored [78]. The addition of these elements influenced the resulting microstructure of AM AA7075. Therefore, further studies should focus on investigating the effect of this microstructure and additional alloying elements on the corrosion resistance of this material.

In general, as for AM Al-Si alloys, the special conditions during additive manufacturing (i.e., highly localized melting and solidification accompanied by extremely high cooling rates) seem to promote a high refinement of the microstructure of these materials and/or the annihilation of certain precipitates/phases. This is generally beneficial in terms of corrosion resistance since it greatly reduces the possible cathodic sites and the galvanic interactions that can promote localized corrosion. Nevertheless, there is a need for further investigations of the corrosion behaviour of other AM Al-based alloys, or Al alloys prepared by MAM techniques other than selective laser melting.

## 5. Summary and Outlook

1. Due to the special conditions associated with the metal additive manufacturing processes, numerous macro- and micro-structural defects can exist within the printed parts. Defects such as porosity, remaining unmolten powder, high surface roughness, and residual stresses, can greatly influence the corrosion performance of these materials. Even though the existence of some of these defects can be limited by carefully tuning the process parameters, they cannot be fully avoided due to the large number of parameters involved and the high complexity of the process. In general, more research should be conducted in order to better understand the influence of these defects and the combined impact of process parameters on the corrosion resistance of these materials.
2. Aside from Al-Si alloys (and specially AlSi10Mg alloy), very limited research has been published concerning the corrosion resistance of other additively manufactured Al alloy parts. More focus should be given to other types of 3D-printed Al-based alloys. Moreover, further research is needed to study the microstructure and corrosion behavior of additively manufactured parts prepared using other metal additive manufacturing methods besides selective laser melting/sintering.
3. In general, most of the published works on additively manufactured Al-based alloys agree that their corrosion resistance is similar to or higher than that of the same alloys fabricated using traditional manufacturing methods.
4. Due to the special conditions associated with the metal additive manufacturing processes (for instance: the use of pre-alloyed metal powder and highly localized (re)melting combined with high cooling rates), a unique microstructure formed by a fine Si network that encloses the α-Al in small cells of varying sizes across the melt pools characterizes the printed Al-Si alloys specimens. A great number of works have already shown that the corrosion behavior of these materials is greatly influenced by this special and unique microstructure. The borders of the melt pools (where larger cells are found) have been identified as vulnerable sites for corrosion to initiate and further propagate. However, in general, the existence of a rather connected Si network for the case of as-built specimens prevents/holds the corrosion from penetrating deeper into the material.
5. The specific (out of equilibrium) microstructure obtained by SLM processing makes the materials susceptible to certain heat treatments (such as stress release and solution T6 heat treatments). Depending on the temperature of the heat treatment, these can disrupt the silicon network in Al-Si alloys, leading to the formation of large and separate Si precipitates. Aside from negatively impacting the mechanical properties, this also affects the corrosion resistance. The disruption of the Si network allows corrosion to penetrate deeper into the material, while larger Si precipitates disrupt the formation of a compact passive layer and form larger cathodic sites that increase the driving force for galvanic corrosion. Additionally, the presence of Mg in most Al-Si alloys makes the material susceptible to precipitation hardening through the formation of Mg_2_Si precipitates. However, the presence of these precipitates can greatly affect the pitting corrosion process in these materials. An alternative to stress release heat treatment is the heating of the building platform during production. In this way, alternation of the microstructure and subsequent change in corrosion behavior can be prevented. However, more research on different building platform temperatures and their effect on the microstructure and corrosion behavior is required. Moreover, further work should focus on adapting existing heat treatments to the specific microstructure of the additively manufactured materials.
6. For additively manufactured Al-Si alloys, increasing the amount of Si, greatly increases the level of connectivity of the silicon network, which reduces the penetration depth of the corrosion attack. However, in these alloys, the presence of small amounts of Mg and Fe cannot be ignored during the analysis of the corrosion behavior.
7. It has been shown that the choice of printing parameters can greatly affect the surface roughness of the printed parts, and thus also their corrosion resistance. The scanning speed, followed by the hatching distance, was reported to have the largest effect. Altering the manufacturing parameters to reduce the surface roughness could also affect the microstructure. In general, an improvement of the corrosion resistance has been reported for samples that underwent post-printing operations to reduce their surface roughness (such as shot peening, sandblasting, and bright dipping) compared to as-produced samples. However, further work is needed to better understand the performance of other post-printing processes (for instance electro-polishing), as well as their effect on the corrosion behavior of additively manufactured Al-based alloys.
8. In general, a limited number of studies have been conducted to understand the mechanisms of corrosion protection of additively manufactured metal parts. Studies on the galvanostatic anodizing of 3D-printed Al-Si alloys demonstrated that the special microstructure of these materials greatly influences the typical voltage-time behavior as well as the characteristics of the anodic oxide layer. A significantly lower oxide growth rate was seen for additively manufactured Al-Si parts compared to cast alloy, attributed to the larger fraction of the anodic charge consumed by the oxidation of the Si phase. The porous anodic oxide film, thinner around the melt pool borders, was characterized by the formation of branched-like pores. The anodizing behavior as well as the anodic oxide layer of additively manufactured Al-Si alloys is greatly affected by defects and special features typical from the metal additive manufacturing process. The intrinsic anisotropy, melt pool borders, and internal pores are among those features affecting the anodization process. More research is still needed to better understand the impact of other defects such as unmolten powder and residual stresses on the anodizing behavior of these materials, as well as to further investigate the properties of the formed anodic oxides. Moreover, additional research is required to understand the anodizing behaviour of these materials under other anodizing conditions such as potentiostatic anodizing regime and different electrolytes.

## Figures and Tables

**Table 1:** Comparison between Cast and as-built AM AlSi10Mg from results obtained in potentiodynamic polarization tests. Only results acquired in polished/ground samples were considered. E_corr_, I_corr_, and E_pit_ refer to corrosion potential, corrosion current density, and pitting potential, respectively.

Reference	Electrolyte	E_corr_	I_corr_	E_pit_	Corrosion Rate
[44]	Aerated 3.5 wt.% NaCl	AM > Cast	Cast > AM		Cast > AM
[51]	Diluted Harrison’s solution	Cast > AM	Cast ~ AM	AM > Cast	
[59]	Aerated 3.5 wt.% NaCl	AM > Cast	Cast > AM		Cast > AM
[60]	Aerated 3.5 wt.% NaCl	AM > Cast	Cast > AM	Cast ~ AM	
[32]	Aerated 0.1 M NaCl	Cast ~ AM	Cast ~ AM		
[61]	Aerated 3.5 wt. %NaCl	Cast ~ AM	Cast ~ AM		Cast ~ AM

**Table 2:** Specification of chemical composition for AA2024 and AA7075.

Material	Al	Si	Fe	Cu	Mn	Mg	Cr	Zn	Ti
**AA2024**	Balance	<0.5	<0.5	**3.8–4.9**	0.3–0.9	1.2–1.8	<0.1	<0.25	<0.15
**AA7075**	Balance	<0.4	<0.5	1.2–2	<0.3	2.1–2.9	0.18–0.28	**5.1–6.1**	<0.2
